# Crystalline-Like Keratopathy after Intravenous Immunoglobulin Therapy with Incomplete Kawasaki Disease: Case Report and Literature Review

**DOI:** 10.1155/2013/621952

**Published:** 2013-03-28

**Authors:** Elif Erdem, Emine Kocabas, Hande Taylan Sekeroglu, Özlem Özgür, Meltem Yagmur, T. Reha Ersoz

**Affiliations:** ^1^Ophthalmology Department, Cukurova University Faculty of Medicine, Balcali Saricam, Adana 01330, Turkey; ^2^Pediatric Infectious Disease Department, Cukurova University Faculty of Medicine, Balcali Saricam, Adana 01330, Turkey; ^3^Ophthalmology Department, Hacettepe Universtiy Faculty of Medicine, Sihhiye, Ankara 06100, Turkey

## Abstract

A 7-year-old girl had presented with high body temperature and joint pain which continued for 3 days. Because of the prolonged history of unexplained fever, rash, bilateral nonpurulent conjunctival injection, oropharyngeal erythema, strawberry tongue, and extreme of age, incomplete Kawasaki disease was considered and started on an intravenous immunoglobulin infusion. Six days after this treatment, patient was referred to eye clinic with decreased vision and photophobia. Visual acuity was reduced to 20/40 in both eyes. Slit-lamp examination revealed bilateral diffuse corneal punctate epitheliopathy and anterior stromal haze. Corneal epitheliopathy seemed like crystal deposits. One day after presentation, mild anterior uveitis was added to clinical picture. All ocular findings disappeared in one week with topical steroid and unpreserved artificial tear drops. We present a case who was diagnosed as incomplete Kawasaki disease along with bilateral diffuse crystalline-like keratopathy. We supposed that unusual ocular presentation may be associated with intravenous immunoglobulin treatment.

## 1. Introduction

Kawasaki disease (KD) is an acute, self-limiting systemic vasculitis of unknown etiology that affects the small- and medium-sized blood vessels of the body, particularly the coronary arteries, which predominantly affects children at 6 months to 5 years of age [1]. It was first described by Kawasaki in 1967 in Japan. KD is diagnosed according to the clinical criteria developed by Kawasaki [2] (Table 1). Some patients who are diagnosed with “incomplete” KD do not fulfill the clinical criteria for classical KD. Such patients are usually at extreme ages and are more at risk for developing an aneurysm of the coronary arteries. Early diagnosis of incomplete KD is vital for timely infusion of intravenous immunoglobulin (IVIG) to prevent coronary complications. When untreated, 15 to 25% of patients develop coronary artery aneurysms [3, 4].

Bilateral nonexudative conjunctival injection is one of the principal clinical features of Kawasaki disease. It typically lasts from 1 to 2 weeks without treatment [5]. Severe ocular complications are uncommon in the disorder. In a recently reported study, 15 patients of 115 patients (13.2%) had ophthalmologic complications, with uveitis in 13, papilledema in one, and conjunctival hemorrhage in another patients [6].

We present a case who was diagnosed as incomplete Kawasaki disease along with bilateral diffuse crystalline-like keratopathy. We supposed that unusual ocular presentation may be associated with intravenous immunoglobulin treatment.

## 2. Case Report

A previously healthy, fully vaccinated 7-year-old girl presented with a 3-day history of high-grade fever (up to 40°C) and generalized joint pain upon admission. According to the clinical history, erythematous rash which began at trunk spread to the entire body and bilateral conjunctival hyperemia developed at the second day of fever.

Physical examination at the time of admission revealed high fever (38°C), oral and pharyngeal erythema, strawberry tongue, erythematous maculopapular rash, and bilateral nonpurulent conjunctivitis. High fever persisted for two days following her admission to the hospital.

Laboratory workup was initiated for suspected infections and rheumatologic causes. White blood cell (WBC) count was 7.11 × 10³/*μ*L with 42% neutrophils, 50% lymphocyte, and 8% monocyte. Erythrocyte sedimentation rate was 15 mm/h, procalcitonin was 0.216 ng/mL (*N* < 0.5), C-reactive protein (CRP) was 115 mg/dL (N < 8 mg/L), anti-streptolysin-O (ASO) was 90 IU/mL (*N* < 200). Red blood cells count and morphology, coagulation parameters were normal but thrombocyte count was near the lower limit 188 × 10³/*μ*L (N: 142 × 10³/*μ*L–424 × 10³/*μ*L) at the beginning of her admission. Serum immunoglobulins (G, A, and M) and C3 values were in normal limits. Blood serological analyses were negative for cytomegalovirus, coxsackie virus, herpes viruses, hepatitis viruses, human immunodeficiency virus, rubella virus, and Epstein-Barr virus. serum antinuclear Antibody (ANA) was negative. There was not any microbiological growth on blood, nasopharyngeal swabs, and urine culture. Electrocardiography and echocardiography were found to be normal.

Because of the prolonged history of unexplained fever, rash, bilateral nonpurulent conjunctival injection, pharyngeal erythema, strawberry tongue, and her extreme age, incomplete Kawasaki disease was considered in this case. The patient presented with three out of five criteria of classic Kawasaki disease (Table 1) and started on an IVIG (2 gr/kg/day) infusion and low-dose aspirin (5 mg/kg/day) at the 2nd day of her admission. Fever, skin lesions, fatigue, and joint pain completely regressed in 4–7 days (Table 2).

**Figure 1:**
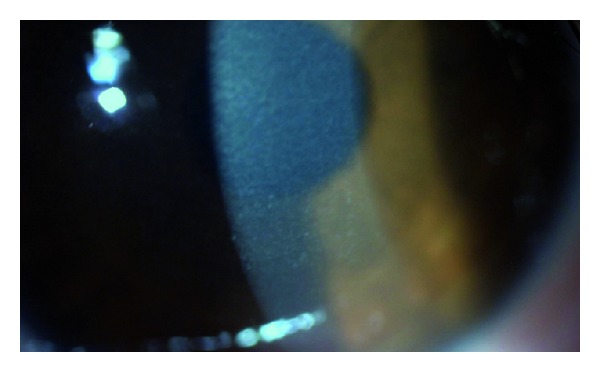
The anterior segment appearance at first examination of the right eye. Diffuse anterior stromal haze and crystalline-like deposits are shown.

**Figure 2:**
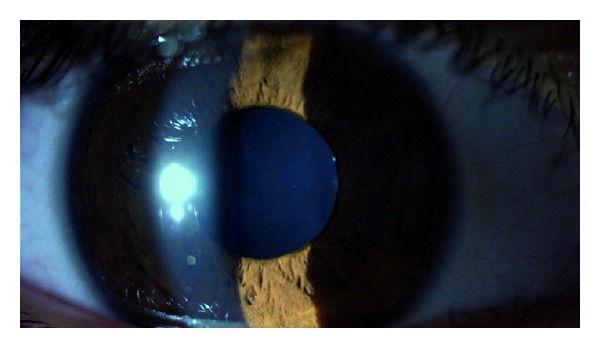
One week after the presentation. Right eye.

**Figure 3:**
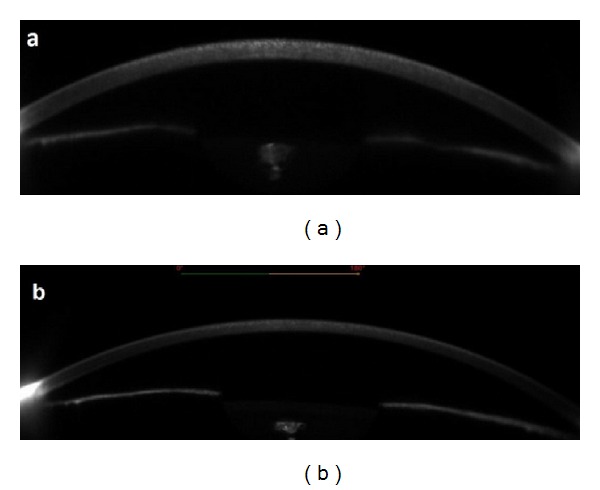
Scheimpflug images. Before and one week after topical steroid treatment. At the bottom picture, decrease of hyperreflective deposits is clearly visible. Right eye.

Six days after the administration of intravenous immunoglobulin treatment, the patient was referred to the eye clinic for decreased vision and severe photophobia. Visual acuity was found to be 20/40 level (at Snellen chart) in both eyes. Slit-lamp examination revealed bilateral diffuse, thin crystalline-like deposits in corneal epithelium and mild anterior stromal haze (Figure 1). The tear film was normal. Punctate staining of corneal epithelium was shown with fluorescein dye. Nonpigmented keratic precipitates and mild (1+) anterior chamber cells were observed at the second day. Fundus examination was normal. There was no previous ocular and/or systemic disease and drug use in clinical history. All of the ocular symptoms and findings disappeared in 1 week with topical steroid (Dexasine SE) and artificial tear drop (Refresh) 4 times a day (Figure 2). Subepithelial hyperreflective deposits were demonstrated at Scheimpflug camera images (Sirius corneal topography, CSO Inc., Italy) of cornea (Figure 3(a) before treatment, Figure 3(b) after treatment). Bilateral visual acuity was 20/20 at the end of the first week.

The patients was discharged on day 11 of hospitalization with aspirin (ASA). High level of acute phase reactants (CRP > 200 mg/dL) and thrombocytosis (538 × 10³/*μ*L–744 × 10³/*μ*L) persisted at the first 15 days and returned to normal at the 21st day.

## 3. Discussion

In this paper, we presented unusual ocular findings of Kawasaki disease. The ocular presentation seemed to be the result of systemic medication or disease because the findings were bilateral and symmetric. Intravenous immunoglobulin therapy was a confounding factor for understanding of the ocular disease pathogenesis.

The various ocular presentations of Kawasaki disease have been reported in the literature are disciform keratitis, optic disc swelling, sudden blindness, superficial punctate keratitis, periorbital vasculitis, and severe global inflammatory involvement of ocular segments [7–11]. The most common ocular findings are conjunctivitis (reported in 95% of patients) and anterior uveitis (reported in 83% of patients) [5, 12]. Fortunately, many of these presentations are self-limited and resolve spontaneously without treatment.

Laboratory tests may be useful for understanding ocular involvement. Ohno et al. found the significant correlations between ocular inflammation and erythrocyte sedimentation rate and C-reactive protein level [7]. Similarly, our case had mild intraocular inflammation, and the blood levels of these markers were high.

To the best of our knowledge, crystalline-like keratopathy in Kawasaki disease was not reported before in the literature. This finding may be associated with immunoglobulin treatment. Corneal diffusion of subconjunctival injected human immunoglobulin has been shown in experimental studies [13]. Indeed, the relation between crystalline keratopathy and immunoglobulin treatment was reported in humans. Budde et al. reported a case with annular crystalline keratopathy, which developed one year following intravenous immunoglobulin treatment. Annular distribution of crystals was characteristic feature of this case [14]. But in our case, distribution of crystalline-like deposits was diffuse.

Systemic diseases (e.g., Tangier disease, tyrosinemia, paraglobulinemia, and cystinosis) certain drugs (e.g., amiodarone, gold- or silver-containing drugs, ergotamine, chloroquine), or Schnyder's and Bietti's corneal dystrophy may cause noninfectious crystalline keratopathy [15–20]. Monoclonal gammopathy related with crystalline keratopathy has been reported [21]. These deposits were described as amorphous material [22]. The immunoglobulins of IgG class was determined at corneal specimens [22]. The present case had no history of the reasons set forth above.

In conclusion, although the pathogenesis of acute onset and steroid-responsive crystalline-like keratopathy in this case is not clear, we can suggest that the intake of exogenous immunoglobulin promoted the development of these findings.

## Figures and Tables

**Table 1:** Classic Kawasaki disease clinical diagnostic criteria.

Clinical criteria	Case patient had bellowing criteria
Fever for ≥5 days plus 4 of the following must be present to make a definitive diagnosis	Yes
Polymorphous rash	Yes
Bilateral conjunctival injections	Yes
At least one of the following:	
(i) Erythema or fissuring of the lip	No
(ii) Strawberry tongue	Yes
(iii) Diffuse injection of oral and pharyngeal mucosa	Yes

Acute, nonpurulent cervical lymphadenopathy (at least one node ≥ 1.5 cm)	No
At least one of the following:	
(i) Erythema of palms and soles	No
(ii) Indurative edema of hands and feet	No
(iii) Membranous desquamation from fingertips	No

**Table 2:** Hospital admission inpatient course.

Symptoms/signs	Day 3 High fever	Day 2 Fever/rash	Day 0 Fever/maculopapular rash	Day 2 Fever/rash	Day 6 Afebrile	Day 11 Discharge
	Joint pain	Conjunctival hyperemia	Bilat. nonpurulent conjunctivitis Pharyngeal hyperemia	Bilat. nonpurulent conjunctivitis	Decreased vision Photophobia	
WBC count, ×10³ cells/*µ*L			7.11			
Hemoglobin, gr/dL			12.3			
Platelet count, ×10³ cells/*µ*L			118	538		744
ESR, mm/h			15			
CRP, mg/dL			115	>200		
ECHO					N	ASA
Treatment					IVIG/ASA	
